# Overall Rebalancing of Gut Microbiota Is Key to Autism Intervention

**DOI:** 10.3389/fpsyg.2022.862719

**Published:** 2022-05-26

**Authors:** Chang Lu, Jiaqi Rong, Changxing Fu, Wenshi Wang, Jing Xu, Xing-Da Ju

**Affiliations:** ^1^School of Psychology, Northeast Normal University, Changchun, China; ^2^School of Life Sciences, Northeast Normal University, Changchun, China

**Keywords:** autism spectrum disorder, gut microbiota, gut-brain axis, prebiotics, microbiota transfer therapy

## Abstract

Autism Spectrum Disorder (ASD) is a neurodevelopmental disorder with unclear etiology, and due to the lack of effective treatment, ASD patients bring enormous economic and psychological burden to families and society. In recent years, many studies have found that children with ASD are associated with gastrointestinal diseases, and the composition of intestinal microbiota (GM) is different from that of typical developing children. Thus, many researchers believe that the gut-brain axis may play an important role in the occurrence and development of ASD. Indeed, some clinical trials and animal studies have reported changes in neurological function, behavior, and comorbid symptoms of autistic children after rebalancing the composition of the GM through the use of antibiotics, prebiotics, and probiotics or microbiota transfer therapy (MMT). In view of the emergence of new therapies based on the modulation of GM, characterizing the individual gut bacterial profile evaluating the effectiveness of intervention therapies could help provide a better quality of life for subjects with ASD. This article reviews current studies on interventions to rebalance the GM in children with ASD. The results showed that *Lactobacillus plantarum* may be an effective strain for the probiotic treatment of ASD. However, the greater effectiveness of MMT treatment suggests that it may be more important to pay attention to the overall balance of the patient’s GM. Based on these findings, a more thorough assessment of the GM is expected to contribute to personalized microbial intervention, which can be used as a supplementary treatment for ASD.

## Introduction

Autism spectrum disorder (ASD) is a group of developmental disorders characterized by impaired social interactions and communication together with repetitive and restrictive behaviors (Hsiao et al., 2013). At present, the diagnostic system for ASD is generally based on the Diagnostic and Statistical Manual of Mental Disorders (5th ed.; DSM-5) and International Classification of Diseases (11th ed.; ICD-11). Epidemiological studies have shown that the prevalence of ASD has been steadily increasing in recent years (Baio et al., 2018; Maenner et al., 2020). Moreover, the difficulty of early diagnosis and the lack of effective therapy method of ASD have brought a great economic burden to society and families (Wang et al., 2018).

Autism Spectrum Disorder (ASD) is multifactorial, mainly including genetic risk factors and environmental risk factors, and the clinical presentation of ASD is highly heterogeneous (Kim and Leventhal, 2015). In recent years, as a special environmental factor, gut microbiota (GM) has gradually attracted people’s attention. Studies have shown that mental and neurological diseases, such as ASD, attention deficit hyperactivity disorder, depression, anxiety disorder, bipolar affective disorder, Parkinson’s disease and Alzheimer’s disease, are related to the imbalance of GM, and are usually accompanied by gastrointestinal (GI) disorders (Naveed et al., 2021). Clinical survey data shows that the risk of GI in ASD children is significantly higher than that in typical developing (TD) children, and the severity of autism is associated with the prevalence of GI (Alabaf et al., 2019). A prospective study has found that gut microbiome at age 1 can predict cognitive performance at age 2, especially in communication behaviors, suggesting a possible correlation between gut microbiome and delayed cognitive or language development (Carlson et al., 2018). Recently, the importance of genes such as *CHD8/chd8*, *Foxp1*, *Slc6a4*, and neuroligin-3 (*Nlgn3*) has been discovered in ASD patients with GI diseases (Niesler and Rappold, 2021). Many studies have proved that the disturbance of the microbiota-gut-brain axis plays an important role in the appearance and development of ASD.

With the importance of GM have been recognized, GM re-balance becomes a potentially effective therapy for ASD children, including oral antibiotics, dietary interventions, probiotics and prebiotics interventions, and fecal microbiota transplantation (FMT). Although these interventions are yielding favorable results in treating autistic behavior-related symptoms, standardized clinical studies will lead to more robust results. In this article, we not only reviewed the possible pathways leading to gut microflora dysbiosis in ASD, but also assessed the potential of gut microbiome in ASD screening. Finally, we evaluated the effects of different therapeutic approaches on GM, aiming to compare the effectiveness of GM rebalancing strategy from the behavioral manifestations, and to explore the correlation between species and behavior. In addition, this study also evaluated the presence of micro markers in ASD patients from the perspective of intervention.

## Gut Dysbiosis in Autism Spectrum Disorder

In addition to neuropsychiatric characteristics, patients with ASD tend to suffer from GI problems. Functional constipation is the most common symptom (Marler et al., 2017), followed by abdominal pain, diarrhea, gas, and vomiting, etc. (Holingue et al., 2018). Many researchers have discussed the complex regulatory relationship (gut-brain axis) between GI system and central nervous system, and there are different views on the relationship between GM and autism (Mayer et al., 2015; Sampson and Mazmanian, 2015; Yap et al., 2021). The main reason for the debate is that the underlying mechanisms of GM affecting the central nervous system is unclear and hard to measure. Nevertheless, the GM still show great potential as non-invasive markers for the diagnosis and therapy of ASD.

### Pathways of Gut Microbiota Affecting Autism Spectrum Disorder

The gut-brain axis indicates that the disorder of host intestinal microbiota may be one of the causes of ASD. Recently, Needham et al. (2020) summarized four approaches to how this “bottom-up” impact is carried out: vagus nerve, stimulation of endocrine cells (including enterochromaffin cells), immune-mediated signaling and transport of gut-derived metabolites from the circulation into the brain. And, they believe that all routes comprising the gut–brain axis are thought to be co-opted by the microbiota to impact brain activity and behavior, and signaling through any one of them may be intertwined with other routes (Needham et al., 2020). Based on these four pathways, this study listed the potential evidence of GM affecting ASD.

(a) The vagus nerve provides a direct neural communication pathway between the GM and the central nervous system (CNS), and promotes the regulation of the GM on the function of CNS. Previous studies have found that toxins produced by *Staphylococcus* and *Bacillus* (staphylococcus enterotoxin and glutenin) can send signals to the brain by stimulating the vagus nerve, so as to induce vomiting or other disease behaviors (Hu et al., 2007). Bravo et al. (2011) found that *Lactobacillus rhamnosus* could reduce anxiety and depression related behaviors only in mice without vagotomy, which further explained that neurotransmitters or other metabolites produced by GM could directly regulate vagal activity by stimulating vagal afferent sensory neurons.

(b) Studies have found that 90% of serotonin in the human body is produced by intestinal chromaffin cells, a secretory cell in the inner layer of the intestine (Gershon and Tack, 2007). Enterochromaffin cell production of serotonin impacts its circulating levels and has the potential to influence brain activity directly or indirectly (De Vedder et al., 2018). In addition, some studies demonstrated that some *Bifidobacterium* and *Clostridium* metabolites can also change the content of serotonin in the intestine (Yano et al., 2015; Tian et al., 2019). Improved performance in mouse models of depression have been shown by probiotic treatment with *Bifidobacterium* spp. in a study that concurrently observed an increase either in the levels of serotonin in the brain or in the secretion of serotonin precursor in enterochromaffin cells *in vitro* (Tian et al., 2019). Moreover, Colonic enterochromaffin cells do express receptors for, and respond to, various microbial metabolites, including microorganism-associated molecular patterns (MAMPs), short chain fatty acids (SCFAs), aromatic amino acid metabolites, and secondary bile acids (Kidd et al., 2008; Reigstad et al., 2015; Tsuruta et al., 2016; Lund et al., 2018).

(c) Studies have proved that there is a correlation between intestinal inflammation and immune dysfunction in ASD patients, such as abnormal balance of T cells in the intestine of ASD patients and increased GI problems in ASD patients (Navarro et al., 2016; Vuong and Hsiao, 2017; Rose et al., 2018). Recently, it has been clearly demonstrated that high concentrations of pro-inflammatory microbiota in the gut can lead to increased intestinal permeability and inflammation, resulting in mild systemic inflammation and immune dysregulation (Felix et al., 2018). In addition, lipopolysaccharide (LPS), as an effective endotoxin in the cell wall of Gram-negative bacteria, has also been proved to induce disease behavior, cognitive impairment and acute depression like behavior in mice by activating systemic inflammation, and affect fetal brain development (Needham et al., 2020). Emanuele et al. (2010) found that the serum LPS level of ASD patients was significantly higher than that of healthy peers and was related to social behavior disorders, which further indicated that immune inflammation may play an important role in the intestinal brain axis.

(d) Many microbial metabolites produced in the gut can pass into systemic circulation at varying levels and rates. One example is SCFAs, where previous studies have shown that any interference in this signal transduction may have a direct impact on the central nervous system, which may lead to neurodevelopmental disorders and neurodegenerative diseases (Borre et al., 2014; Hill et al., 2014). Moreover, it has been proved that SCFAs metabolized by intestinal microorganisms can enter the circulatory system to regulate immune and inflammatory reactions, and then affect the neural function and development of human brain (Foley et al., 2014; Frost et al., 2014; Chambers et al., 2015). In spite of many studies support the health benefits of SCFAs, such as energy supply for epithelial cells, restoring epithelial barrier function, anti-inflammatory, and immunomodulating activities (Richards et al., 2016). However, it is important to note that excessive quantities of propionic acid (the main SCFA produced by *Clostridium*, *Bacteroides*, and *Desulfovibrio*) have been reported in irritable bowel syndrome, and necrotizing enterocolitis (Wang et al., 2007; Tana et al., 2010). In addition, the study found elevated levels of SCFA in the feces of children with autism (Wang et al., 2014). Although it needs to be established whether these elevated intestinal levels of SCFA are high enough to reach substantial levels in the brain, studies in rats have shown that exposure to propionic acid leads to significant deterioration of social behavior, which has shown that propionic acid may has harmful effects on neurological function (Thomas et al., 2012; Foley et al., 2014).

### Potential of Gut Microbiome for Screening of Autism Spectrum Disorder

Children with ASD face the problem of difficult early diagnosis. Most children show some abnormal behavior symptoms only at about 18–24 months, while other specific functional characteristics may only be found at an older age (Borghi and Vignoli, 2019). Current studies indicate that there are multiple subtypes of ASD, potentially caused by different routes of pathophysiology and each with diverse comorbid psychiatric and medical conditions (e.g., gastrointestinal symptoms, allergies, sleep disorders) (Huang et al., 2021). However, this heterogeneity is not addressed by the conventional DSM5-based behavioral diagnostic criteria. Accordingly, it is particularly essential to discover effective objective physiological indicators as the basis for clinical diagnosis and evaluation of ASD. In the past decade, as the importance of GM in ASD has been identified, researchers have begun to investigate the microbial diversity of ASD patients, seeking to identify certain gut microbial characteristics as biomarkers for ASD. Unfortunately, the results of two recent meta-analyses show that these cohort studies have produced inconsistent results in exploring the intestinal microbiota of ASD children (Xu et al., 2019; Iglesias-Vazquez et al., 2020). The interactions between ASD and GM may be influenced by complicated factors such as genetic background, daily diet and the physiological status of the host, which may explain the conflicting results of these studies. However, it is worth noting that most studies have found that the overall diversity of GM (composed of archaea, bacteria, fungi, and viruses) in ASD children increases, while its fungal diversity decreases (Kuehbacher et al., 2006; Finegold et al., 2010; Zou et al., 2021). This suggests that there may be too many harmful bacteria in ASD children, such as *Clostridium* and *Desulfovibrio*, which are more common in ASD patients, also considered to be potential pathogenic bacteria of ASD (Parracho et al., 2005; Finegold, 2011).

In order to verify the claims on previous research concerning changes in the gut microbiome associated with ASD, Wu et al. (2020) performed Machine-learning based on feature selection and classification evaluation which were performed in the training cohort, the validation cohort, and independent diagnosis cohorts to evaluate the potential of the gut microbiome as a non-invasive biomarker for ASD. The results showed that *Prevotella*, *Roseburia*, *Ruminococcus*, *Megasphaera*, and *Streptococcus* may be potential biomarkers of ASD, especially *Prevotella* has significant differences between ASD patients and typical neurodevelopers (Wu et al., 2020), but this result is not consistent with the prediction model established by Zhai et al. (2019). One possible reason for this inconsistency is that the composition of intestinal microbiota is affected by the *in vivo* and *in vitro* environmental factors of its host individual, and the other influence could be the calculation method used in establishing the prediction model. Besides, the quality control conditions and methods of sequencing data might affect the results of the prediction model as well. Therefore, further studies may be required to explore the GM characteristics of ASD. In addition, previous studies mainly focused on the differences of GM between ASD patients and normal people, but rarely analyzed the changes of these biomarkers from the perspective of intervention. Thus, this review discusses different treatment methods, compares the changes of intestinal flora before and after intervention, and further looks into whether intestinal flora has great potential in ASD screening.

## Intervention Method of Autism Spectrum Disorder Children Based on Gut Microbiota

Nowadays, internationally approved and recommended ASD therapies include rehabilitation, education and psychotherapy. In addition, many alternative therapies have been tested, including antibiotics, probiotics, dietary intervention and gut microflora transfer therapy.

### Antibiotics and Dietary Interventions

Although much research has shown that antibiotics can improve the GI and behavioral symptoms of ASD children, there are still some disputes about antibiotic treatment. In principle, antibiotics not only kill potentially harmful bacteria, but also kill beneficial bacteria in ASD patients, thus increasing the probability of GI diseases in ASD children (Vargason et al., 2019). Therefore, antibiotic therapy may not be an optimal intervention for GM rebalancing.

Recently, dietary interventions in children with ASD are very popular. Previous studies have shown that a simple, light and nutritious Mediterranean diet impacts the GM and associated metabolome as well as cardiovascular diseases and neurobehavioral health outcomes (Atladottir et al., 2012; Liu et al., 2017). Therefore, we summarized the studies of dietary intervention in ASD (Table 1). Many studies have shown that the ability of a ketogenic diet (KD, i.e., a high fat diet that has demonstrated beneficial effects on mitochondrial dysfunction and epilepsy) to mitigate some of the neurobehavioral symptoms associated with ASD in an animal model (Verpeut et al., 2016; Castro et al., 2017). Improvements in seizure control and neurobehavioral symptoms have also been reported in ASD children with mild-moderate types of ASD as a result of following a KD (Evangeliou et al., 2003; Herbert and Buckley, 2013; El-Rashidy et al., 2017; Lee et al., 2018; Żarnowska et al., 2018). In addition, the gluten-free and casein-free (GFCF) diet is also one of the most popular dietary therapies for ASD. Some publications report favorable results in the core or peripheral symptoms of autism after a GFCF diet: communication and language, social interaction, stereotyped behavior, hyperactivity, and gastrointestinal symptoms (Knivsberg et al., 2002; Elder et al., 2006; Whiteley et al., 2010; Johnson et al., 2011; Pennesi and Klein, 2012; Herbert and Buckley, 2013; Navarro et al., 2015; Ghalichi et al., 2016; El-Rashidy et al., 2017). However, data on the efficacy of a GFCF diet as a treatment for ASD in children are limited (Pusponegoro et al., 2015; Hyman et al., 2016; Gonzalez Domenech et al., 2019; Josw Gonzalez-Domenech et al., 2020; Piwowarczyk et al., 2020). Particularly in recent years, there have been many reports of an absence of behavioral improvement after such diets. Even recently, researchers have shown that dietary interventions could potentially have a harmful effect (Fattorusso et al., 2019). For example, restrictive diets further limit the variety of food intake since individuals with ASD already exhibit picky eating behavior, so restrictive diets can result in macronutrient and micronutrient deficiencies. Moreover, the food taken by this kind of diet method is usually expensive, which imposes an additional burden on the families of ASD children, and the standard of dietary intervention is extremely strict and does not apply to all ASD patients.

**TABLE 1:** Dietary intervention studies.

Authors	Study design	Treatment	Effect on behavioral symptoms	Effect on GI symptoms
Knivsberg et al. (2002)	A randomized controlled trial of 20 ASD children aged 5–10 with abnormal urinary peptide levels	GFCF diet vs. RD for 12 months	The GFCF diet group improved more in LIPC, and the peer relationship and language communication were also better improved	−
Evangeliou et al. (2003)	A pilot prospective follow-up study on 30 children with autistic behavior from 4 to 10 years old	KD for 6 months	18 of 30 children (60%), improvement was recorded in several parameters and in accordance with the CARS	−
Elder et al. (2006)	A randomized, double blind repeated measures crossover design on 15 children with autism from 2 to 16 years old	GFCF diet for 6 weeks + RD for 6 weeks	Improvement of their language skills and reducing excessive tension and irritability, but the scores of CARS (*P* = 0.85) and ECOS (*P* = 0.29) decreased and the improvement of behavioral frequencies was not significant	−
Whiteley et al. (2010)	A randomized, controlled trial on 72 children with autism from 4 to 10 years old	GFCF diet vs. RD for 12 months	A significant improvement to mean diet group scores (time × treatment interaction) on sub-domains of ADOS, GARS and ADHD-IV measures	−
Johnson et al. (2011)	A prospective, open label, randomized, parallel groups design on 22 children with autism from 3 to 5 years old	GFCF diet vs. Healthy Control Diet vs. Omega 3 supplementation, for 3 months	Both treatment groups evidenced some gains across a range of variables, including measures of behavior, language, and ratings of the core features of ASD [in Mullen scales of early learning and CBCL]. No statistically significant differences were noted between treatment groups	−
Pennesi and Klein (2012)	A questionnaire analysis study on 387 children with ASD	The questionnaire survey of GFCF diet of ASD children	Improvement of their behavior symptoms, physiological symptoms and social behavior	
Herbert and Buckley (2013)	A case report of a child with autism and epilepsy	GFCF diet, then KD for 14 months	Improvement cognitive and social skills, language function, and stereotypies and reached seizure-free status	−
Navarro et al. (2015)	A randomized double-blind, placebo-controlled study on 12 children with autism from 4 to 7 years old	2 weeks of GFCF diet followed by 4 weeks of GFCF diet + supplement containing brown rice flour	Decrease in Inattention of CBCL-R; improvement in Irritability of ABC and Hyperactivity of ABC and CBCL-R	−
Pusponegoro et al. (2015)	A randomized, controlled, double-blind trial was performed on 74 children with ASD with severe maladaptive behavior and increased urinary I-FABP	Gluten–casein vs. placebo for 7 days	Administrating gluten–casein to children with ASD for 1 week did not increase maladaptive behavior	GI symptom severity did not increase
Hyman et al. (2016)	A case of 22 children with autism from 3 to 4 years old	GFCF diet vs. RD, for 18 weeks + Challenges occurred once per week for 12 weeks	Not find evidence of benefit from the GFCF diet	−
Ghalichi et al. (2016)	A randomized clinical trial, 80 children diagnosed with ASD from 4 to 16 years old	GFD vs. RD for 6 weeks	According to the scores of ADI-R, CARS-2 and, GFD intervention significantly decreased behavioral disorders and prevalence of gastrointestinal symptoms (*P* < 0.05)	Decrease in ROME III questionnaire scores, GI symptoms improved
El-Rashidy et al. (2017)	A case-control study on 45 children with ASD from 3 to 8 years old	KD vs. GFCF vs. RD, for 6 months	Both diet groups showed significant improvement in ATEC and CARS scores, KD group scored better results in cognition and sociability compared to GFCF diet group	-
Lee et al. (2018)	Cohort study of 15 children ages 2–17 years	Modified ketogenic gluten-free diet regimen with supplemental MCT for 3 months	Improved core autism features assessed from the ADOS-2	−
Żarnowska et al. (2018)	A case report of clinical on a 6 years child with autism	KD for 16 months	The patient’s behavior and intellect improved (in regard to hyperactivity, attention span, abnormal reactions to visual and auditory stimuli, usage of objects, adaptability to changes, communication skills, fear, anxiety, and emotional reactions)	−
Gonzalez Domenech et al. (2019)	A crossover clinical trial on 28 children with ASD	3 months GFCF diet + 3 months RD	Not find evidence of benefit from the GFCF diet	−
Piwowarczyk et al. (2020)	A randomized, controlled, single-blinded trial on 66 children with ASD from 3 to 6 years old	GFD vs. GD for 6 months	A GFD compared with a GD did not affect functioning of children with ASD	−
Josw Gonzalez-Domenech et al. (2020)	A crossover trial on 37 children with ASD	6 months GFCF diet + 6 months RD	No significant behavioral changes after GFCF diet	

*I-FABP, Intestinal Fatty Acids Binding Protein; GFCF, Gluten-Free and Casein-Free; RD, Regular Diet; KD, Ketogenic Diet; Challenges, foods that contained gluten only, casein only, both gluten and casein, or neither (placebo); MCT, Medium-Chain Triglycerides; GFD, Gluten Free Diet; GD, Gluten Diet; LIPS, Leiter International Performance Scale; CARS, Childhood Autism Rating Scale; ECOS, Ecological Communication Orientation Scale; ADOS, Autism Diagnostic Observation Schedule; GARS, Gilliam Autism Rating Scale; ADHD-IV, Attention-Deficit Hyperactivity Disorder-IV scale; CBCL, Child behavior checklist; CBCL-R, Conners’ Parent Rating Scale-Revised; ABC, Autism Behavior Checklist; ADI-R, Autism Diagnostic Interview-Revised; ATEC, Autism Treatment Evaluation Checklist; GI, gastrointestinal.*

### Probiotic and Prebiotic Interventions

Probiotics are defined as live microorganisms that, when administered in adequate amounts, benefit the host’s health. Prebiotics refer to non-digestible fibers, such as oligosaccharides, that promote growth and improve the functioning of the probiotics in the GI tract by acting as a specific substrate. Initial evidence suggests that supplementing probiotics and prebiotics may have a good preventive effect on neurological and mental diseases such as Alzheimer’s disease, Parkinson’s disease, depression, and autism spectrum disorder (Yang et al., 2021). Moreover, some research has discovered that since some common abnormal genes between ASD and GI diseases, the abnormal genes may cause abnormal GM in ASD patients (Niesler and Rappold, 2021). Considering the two-way communication of gut brain axis, we believe that probiotic intervention in ASD infants may affect the expression of related genes and decrease the prevalence of ASD. However, the specificity of GM in different patients suggests that precision medicine may be the hope of the future, where treatment protocols will be tailored for specific subpopulations of patients. Therefore, in order to explore the effectiveness of different probiotic and prebiotic therapies on behavioral symptoms and GI symptoms of ASD patients, we summarized the existing probiotic and prebiotic interventions, which can be divided into single strain intervention (Table 2), mixed strain intervention (Table 3), single probiotic and probiotic plus prebiotic intervention (Table 4).

**TABLE 2:** Single probiotic intervention studies.

Authors	Study design	Treatment	Effect on gut microbiota	Effect on behavioral symptoms	Effect on GI symptoms
Parracho et al. (2010)	Randomized double-blind placebo-controlled study on children with ASD from 3 to 16 years old	*Lactobacillus plantarum* WCSF1 vs. placebo, for 12 weeks	*Lactobacilli* and *Enterococci* increased significantly, the count of *Clostridium cluster* XIVa decreased significantly, and there was no significant difference in Chis150 (*Clostridium clusters* I and II)	Decrease in TBPS and DBC scores, in which the scores of disruptive antisocial behaviors, anxiety, self-focused behaviors and communication problems in probiotic group are lower than the baseline	GI symptoms improved
Kaluzna-Czaplinska and Blaszczyk (2012)	Cohort study of children with ASD from 4 to 10 years old	*L. acidophilus* Rosell-11 for 2 months	−	Improvement in their ability to concentrate and fulfill orders, with no impact on behavioral responses to other people’s emotions or eye contact	–
Partty et al. (2015)	Randomized trial, placebo-controlled study on infants followed for 13 years	*L. rhamnosus* GG vs. placebo for the first 6 months of life	At the 6th month, the count of *Bifidobacteria* in children with ADHD / ASD was significantly lower than that in healthy children; At 18 months, the count of *Bacteroides* and *Lactobacillus*-*Enterococcus* group decreased; At 24 months, the count of *Clostridium histolyticum* group decreased	At the age of 13 years, 6 out of 35 (17.1%) children in the placebo group were diagnosed with ASD or ADHD, but none in the probiotic group	–
Liu et al. (2019)	Randomized, double-blind, parallel, placebo-controlled study of 71 patients with ASD aged 7–15 years	*L. plantarum* PS128 vs. placebo for 4 weeks	–	Decrease in CGI-S, CGI-I, ABC-T, SRS, CBCL and SNAP-IV scores, and anxiety, hyperactivity, rule violation, impulse and antisocial behavior were improved	–
Kong et al. (2021)	Randomized, double-blind, placebo-controlled study of 35 ASD patients aged 3–20 years	*L. plantarum* PS128 vs. placebo for 28 weeks, and from the 16th week, both groups received oxytocin	*Roseburia*, *Streptococcus* and *Veillonella* were observed only in the probiotic group, and the content of serum OXT (oxytocin) decreased in the probiotic group	Decrease in ABC, SRS and CGI scores, and irritability and cognitive ability were improved	–

*TBPS, Teacher Beliefs and Practices Scale; DBC, Developmental Behavior Checklist; CGI, Clinical Global Impressions; CGI-S, Clinical Global Impression-Severity; CGI-I, Clinical Global Impression-Improvement; ABC-T, Autism Behavior Checklist-Taiwan version; SRS, Social Responsiveness Scale; SNAP-IV, Swanson, Nolan, and Pelham-IV; CBCL, Child behavior checklist; ABC, Autism Behavior Checklist; GI, gastrointestinal.*

**TABLE 3:** Mixed probiotic intervention studies.

Authors	Study design	Treatment	Effect on gut microbiota	Effect on behavioral symptoms	Effect on GI symptoms
Tomova et al. (2015)	Real-time PCR on fecal samples of 10 children with autism before and after probiotic administration	A mixture of *Lactobacilli*, *Bifidobacteria* and *Streptococci* given 3 times a day for 4 months	Normalization of *Bacteroides/Firmicutes* ratio and *Desulfovibrio spp.* abundance; the count of *Bifidobacterium* and *Desulfovibrio* decreased significantly, and the absolute amount of *Lactobacillus* decreased (relative amount increased), reaching the level of healthy subjects	Decrease in ADI scores, and restrictive and stereotyped behavior were improved	−
Grossi et al. (2016)	Case report of a 12 years old boy with ASD and severe cognitive disability	A mixture of *Bifidobacteria*, *Lactobacilli* and *Streptococci* given daily for 4 weeks	−	Decrease in AODS-2 scores, and social behavior and neurosexual behavior were improved	GI symptoms improved
Shaaban et al. (2018)	Cohort study of 30 children with ASD from 5 to 9 years old	*L. acidophilus*, *L. rhamnosus* and *B. longum* for 3 months	The count of *Bifidobacteria* and *Lactobacillus* increased significantly	Decrease in ATEC scores, and speech/language communication, sociability, sensory/cognitive awareness and health/physical/behavior were improved	Decrease in 6-GSI scores
West and Roberts (2013)	Cohort study of 33 children with ASD	Delpro *^a^* and Del-Immune V *^b^*, for 21 days	−	Decrease in ATEC scores in 88% of children, and speech/language communication, sociability, sensory/cognitive awareness and health/physical/behavior were improved	Improvement of constipation and diarrhea, and GI symptoms improved
Arnold et al. (2019)	Randomized, double-blind, parallel, cross-controlled study of 10 ASD patients aged 3–12 years	Two groups were randomly assigned to receive 8 weeks each on VISBIOME*^c^* and placebo separated by a 3-week washout	The relative abundance of *Ruminococcaceae* and *Bifidobacteriaceae* decreased, and the relative abundance of *Bacteroidaceae* and *Verrucomicrobiaceae* increased, but not significant	Decrease in ABC, CSHQ, PRAS-ASD, PSI and SRS scores, and the sleep problems were significantly improved	Decrease in PedsQL GI scores, and GI symptoms improved
Santocchi et al. (2020)	Randomized, double-blind, parallel, placebo-controlled study of 63 patients with ASD aged 18–72 months	Vivomixx^®^ *^d^* vs. placebo, two pack once a day for the first month; Vivomixx^®^ vs. placebo, one pack once a day for the next 5 months	−	Decrease in ADOS-CSS scores, but not significantly; decrease in ADOS-CSS and VABS II scores in non-GI, with social and communication skills significantly improved	Decrease in 6-GSI scores, and GI symptoms improved

*^a^Delpro^®^ is a probiotic supplement, including Lactobacillus acidophilus, Lactobacillus casei, Lactobacillus delbrueckii, Bifidobacterium longum and Bifidobacterium bifidum.*

*^b^Del-Immune V^®^ is a supplement containing stem fermentation cell lysate and DNA fragment of probiotic strain Lactobacillus rhamnosus V (DV strain).*

*^c^VISBIOME (VSL#3) is a probiotic supplement, including four strains of Lactobacilli (L. casei, L. plantarum, L. acidophilus and L. delbrueckii subsp. bulgaricus), three strains of Bifidobacteria (B. longum, B. infantis, and B. breve), one strain of Streptococcus thermophilus, and starch.*

*^d^Vivomixx^®^ is a probiotic supplement, and each packet contained 450 billions of eight probiotic strains, including Streptococcus thermophilus, Bifidobacterium breve, Bifidobacterium longum, Bifidobacterium infantis, Lactobacillus acidophilus, Lactobacillus plantarum, Lactobacillus paracasei, Lactobacillus delbrueckii subsp. Bulgaricus. ADI, Autism Diagnostic Interview; AODS-2, Autism Diagnostic Observation Schedule-2; CSHQ, Children’s Sleep Habits Questionnaire; PRAS-ASD, Parent-Rated Anxiety Scale for ASD; PSI, Parenting Stress Index; ADOS-CSS, Autism Diagnostic Observation Schedule – Calibrated Severity Score; VABS II, Vineland Adaptive Behavior Scales-Second Edition; 6-GSI, 6-Gastrointestinal Severity Index; PedsQL, Pediatric Quality of Life Inventory; ABC, Autism Behavior Checklist; ATEC, Autism Treatment Evaluation Checklist; GI, gastrointestinal; SRS, Social Responsiveness Scale.*

**TABLE 4:** Intervention studies of prebiotics and probiotics combined with prebiotics.

Authors	Study design	Treatment	Effect on gut microbiota	Effect on behavioral symptoms	Effect on GI symptoms
Grimaldi et al. (2018)	Randomized, double-blind, placebo-controlled study of 30 ASD patients aged 4–11 years	B-GOS, for 6 weeks	The diversity of gut microbiota increased, but there was no significant difference; the relative abundance of *Bifidobacterium* and *Veillonellaceae* decreased, while the relative abundance of *Faecalibacterium prausnitzii* and *Bacteroides* increased	Decrease the antisocial behavior score in ATEC; improve the sleep quality score in SCAS-P; decrease in AQ scores only for ASD patients on a restricted diet (gluten free casein free diet)	GI symptoms improved
Inoue et al. (2019)	Cohort study of 13 ASD patients aged 4–9 years	6 g PHGG every day, for 2 months or more	The relative abundance of *Acidaminococcus* and *Blautia* increased, while the relative abundance of *Streptococcus, Odoribacter* and *Eubacterium* decreased	Decrease the irritability subscale score in ABC-J	GI symptoms improved
Sanctuary et al. (2019)	Randomized, double-blind, cross-controlled study of 8 ASD patients with GI aged 2–11 years	Two groups were randomly assigned to receive 5 weeks each on the BCP alone and the combination of *Bifidobacterium infantis* and BCP separated by a 2-week washout	−	Decrease in ABC scores, especially when stereotyping behavior and sleep problems were improved, but the improvement was more pronounced when prebiotics were taken alone	Decrease in QPGS-RIII and GIH scores, and GI symptoms improved
Wang et al. (2020)	Randomized, double-blind, placebo-controlled study of 26 ASD patients aged 3–9 years	The combination of probiotics①and FOS vs. placebo, for 108 days	The relative abundance of *Bifidobacteriales* and *B. longum* increased, while the relative abundance of some harmful bacteria decreased, such as *Clostridium* and *Ruminococcus*	After the first 30 days, decrease in ATEC scores, but not significant; after the 30–60 days, decrease significant in ATEC scores	Decrease significant in 6-GSI scores, and GI symptoms improved

*B-GOS^®^, Bimuno^®^ galactooligosaccharide; PHGG, Partially hydrolyzed guar gum; BCP, Bovine colostrums powder; Probiotics①, is a probiotic blend, including Bifidobacterium infantis Bi-26, Lactobacillus rhamnosus HN001, Bifidobacterium lactis BL-04 and Lactobacillus paracasei LPC-37; FOS, fructooligosaccharide; SCAS-P, Spence’s Children Anxiety Scale-Parent version; AQ, Autism Spectrum Quotient; ABC-J, Aberrant Behavior Checklist, Japanese Version; QPGS-RIII, Questionnaire on Pediatric Gastrointestinal Symptoms-Rome III Version; GIH, Gastrointestinal History; ABC, Autism Behavior Checklist; ATEC, Autism Treatment Evaluation Checklist; GI, gastrointestinal; 6-GSI, 6-Gastrointestinal Severity Index.*

#### Single Strain Interventions

The strains used in single strain intervention mainly come from *Lactobacillus*. Parracho et al. (2010) previously found that taking *Lactobacillus plantarum* WCSF1 significantly increased the number of *Lactobacillus* and *Enterococcus* bacteria in the intestines of children with ASD, and significantly reduced the count of *Clostridium cluster* XIVa, a harmful bacterium. Moreover, after probiotic intervention, the scores of destructive behavior, anxiety, self-focused behavior and communication disorder in developmental behavior checklist (DBC) scale of ASD children were lower than the baseline level (Parracho et al., 2010). Kaluzna-Czaplinska and Blaszczyk (2012) found that *L. acidophilus* Rosell-11 can upgrade the ability of ASD children to concentrate and complete commands, but unlike *Lactobacillus plantarum* WCSF1, it does not affect the emotional or eye contact response of ASD children in social interaction. A similar conclusion was also reached by the Partty’s research. By randomly giving 75 newborn infants *L. rhamnosus* GG (LGG) or placebo for 6 months, they found that after 13 years, attention deficit hyperactivity disorder (ADHD) or Asperger syndrome (AS) was diagnosed in 6/35 (17.1%) children in the placebo and none in the probiotic group. It can be seen that LGG plays an important role in the development of children’s attention (Partty et al., 2015). Recently, *Lactobacillus plantarum* PS128 has also been proved to be effective in ASD intervention. Both cohort research found that taking *Lactobacillus plantarum* PS128 could reduce the scores of ASD children on the social responsiveness scale (SRS) and clinical global impressions (CGI) scale. In other words, *Lactobacillus plantarum* PS128 can improve the irritability, anxiety, hyperactivity, cognition, ring breaking behavior and communication behavior of ASD children (Liu et al., 2019; Kong et al., 2021). Moreover, Kong et al. (2021) also found that the combination of *Lactobacillus plantarum* PS128 and serum oxytocin (OXT) showed a better effect in the treatment of ASD. In conclusion, these results suggest that a single strain (mainly *Lactobacillus*) can batter the symptoms of ASD to a certain extent, and there are similar conclusions in the study of mice. For example, recently researchers discovered that *Lactobacillus reuteri* can batter the anxiety and stereotyped behavior of Cntnap2 KO mice (an animal model of ASD) (Bellone and Luscher, 2021). However, it is worth noting that these strains do not show a consistent conclusion on the impact of these strains on the GM of ASD children, so it is difficult to explain the relationship between the improvement of behavioral symptoms and the regulation of GM balance.

#### Mixed Strain Interventions

Recently, many interventions no longer limited to a single strain, but mixed lactobacillus with *Bifidobacterium* and/or *Streptococcus* to intervene in ASD children. For example, Shaaban et al. (2018) found that after oral administration with the mixture of *Lactobacillus acidophilus*, *Lactobacillus rhamnosus* and *Bifidobacterium longum*, besides the colony count of *Bifidobacteria* and *Lactobacillus* in their intestinal tract increased, those ASD children had lower scores in the autism treatment evaluation checklist (ATEC), indicating that the verbal communication and social ability of children with ASD improved. Interestingly, this improvement in behavioral symptoms was also demonstrated in two intervention studies in which ASD patients were treated with a mixture of *Lactobacillus*, *Bifidobacteria*, and *Streptococci* (Tomova et al., 2015; Grossi et al., 2016). However, Tomova et al. (2015) found that probiotics significantly reduced the number of *Bifidobacteria* and *Lactobacillus* in the gut of ASD patients. Therefore, similar to the results of single strain intervention, probiotics mixed reagent has different effects on the GM of ASD patients, but it is worth noting that both of them can increase the relative abundance of *Lactobacillus* in the gut of ASD patients. In addition, probiotic supplements such as Delpro^®^, Vivomixx^®^, and VISBIOME (VSL#3) have been used to intervene in ASD patients with improved behavioral symptoms, especially verbal communication and social behavior, and VISBIOME even improved sleep quality and life quality of ASD patients (West and Roberts, 2013; Arnold et al., 2019; Santocchi et al., 2020). Nevertheless, the effectiveness demonstrated by these probiotic supplements did not show an advantage over the single *Lactobacillus* intervention. For example, although each package of VISBIOME probiotic supplement has a higher dose of bacteria than the single strain intervention used by Liu et al. (2019) (9 × 10^12^ CFUs vs. 3 × 10^10^ CFUs), it can be seen only from the SRS score before and after the intervention that the improvement effect of VISBIOME probiotic supplement on ASD children is not better than that of *Lactobacillus plantarum* PS128 when the intervention duration is 4 weeks (Arnold et al., 2019). In the study of Kong et al. (2021), it also proved that the improvement effect of single *Lactobacillus plantarum* PS128 on the scores of Irritability (S1), Social Withdrawal (S2), and Stereotypic Behavior (S3) in Autism Behavior Checklist (ABC) scale was better than that of VISBIOME, but this could not rule out the reason that the experimental intervention cycle of Kong et al. (2021) was longer. In addition, there is little evidence that taking probiotic mixed reagents can reduce the anxiety of ASD. Beyond that, few researchers have investigated whether there is synergistic or antagonistic effect of different strains in these mixed reagents in the gut of ASD patients. Moreover, at present, there is no standardized intervention cycle and dose for probiotic intervention, and researchers do not use a unified behavior scale for the detection of behavioral symptoms of ASD children. This brings great difficulty to the comparison of the effectiveness of different probiotic interventions. But anyway, it is certain that these interventions are at least harmless, and *Lactobacillus* is beneficial to ASD patients.

#### Single Probiotic and Probiotic + Prebiotic Interventions

Some researchers started to consider the overall balance of the GM ecosystem of ASD patients, and put forward the intervention therapy of the probiotics and the combination of probiotics and prebiotics. Grimaldi et al. (2018) found that the social behavior and sleep quality of ASD children were improved by giving 30 ASD children Bimuno^®^ galactooligosaccharide (B-GOS^®^) prebiotic reagent for 6 weeks. Inoue et al. (2019) found that partially hydrolyzed guar gum (PHGG) improved irritability in ASD patients. These evidence suggested that the administration of prebiotics can cause the probiotics in the gut to generate specific metabolites, which is of great significance in balancing the entire GM ecosystem and treating ASD (Davies et al., 2021). Recently, a combination of probiotics and probiotics has been administered to ASD patients with good results. For example, after 1 month of constant supplementation of probiotics (*Bifidobacterium infantis* Bi-26, *Lactobacillus rhamnosus* HN001, *Bifidobacterium lactis* BL-04, and *Lactobacillus paracasei* LPC-37) and fructooligosaccharide (FOS, growth factors of *Bifidobacterium*) in ASD patients, there ATEC total score continuously decreased over 2 months, especially in which the scores of speech/language/communication and social interaction decreased significantly, indicating improvements in verbal communication and social behavior of those autism patients (Wang et al., 2020). Moreover, consistent with the results of previous studies, taking probiotics and FOS mixed supplements can increase the count of *Bifidobacteria* and *B. longum* in the intestine of ASD patients and decrease the amount of some harmful bacteria such as *Clostridium* and *Ruminococcus*. Sanctuary et al. (2019) found that although the total ABC scale score decreased for ASD patients receiving intervention therapy, along with improved stereotyped behavior and decreased sleepiness, the changes of GM varied from person to person. Interestingly, they also found that bovine colostrum powder (BCP) alone could improve the behavioral symptoms of ASD patients more significantly than the combination of *Bifidobacterium infantis* and BCP (Sanctuary et al., 2019). This result convinces that the overall balance of intestinal micro ecosystem is more important than the change of single strain. Recently, a meta-analysis demonstrated that prebiotics and probiotic-containing probiotics performed better than probiotic interventions in the treatment of ASD (Davies et al., 2021), further supporting our view that microbium-based interventions should focus on the overall balance of the patient’s intestinal microecology.

In summary, we found that although probiotics and probiotics intervention showed certain effects in improving ASD behavioral symptoms, there were obvious differences in their effects on GM of ASD, and it was still difficult for researchers to give specific explanations on the biological mechanism of how probiotics and probiotics affected ASD behavior. In addition, alterations in gut microbiome composition have been confirmed in children with ASD, but few probiotics and prebiotics interventions have been designed for the gut microbiome characteristics of ASD. The effectiveness of prebiotic and mixed probiotic intervention compared with probiotic alone also shows that the overall balance of GM in ASD patients may need more attention during the intervention process.

### Fecal Microbiota Transplant Therapy

Fecal microbiota transplant (FMT), consisted of transferring the fecal microbiota from healthy volunteers to patients with gut dysbiosis, may alleviate GI and neurobehavioral symptoms in children with ASD by rebalancing the physiological intestinal microbiota. We have summarized these studies in Table 5. Linda et al. (2016) used FMT to intervene ASD, and found that behavioral symptoms and GI symptoms improved in younger ASD patients, while there was no significant change in older patients (21 years old) before and after the intervention, proving the feasibility of FMT for the treatment of children with ASD. Based on this, Zhao et al. (2019) conducted a randomized controlled study of 48 patients with ASD. The FMT group received two FMT treatments (2 months apart) and the control group received only rehabilitation training. They found that after the first FMT treatment, the Childhood Autism Rating Scale (CARS) scores in the FMT group decreased by 10.8% (behavioral symptoms of ASD improved) compared with 0.8% (*P* < 0.001) in the control group. After the second FMT, the CARS scores in the FMT group continued to decrease slightly (*P* = 0.074), further demonstrating the efficacy of FMT. However, there are still some problems with FMT. For example, 7 cases (29.2%) in the FMT group had adverse reactions such as fever, allergy and nausea during the intervention. Therefore, some argue that the feasibility of this approach for all ASD children needs further validation.

**TABLE 5:** Intervention studies of FMT and MTT.

Authors	Study design	Treatment	Effect on gut microbiota	Effect on behavioral symptoms	Effect on GI symptoms
Linda et al. (2016)	Cohort study of 9 ASD patients (2, 3, 5, 5, 6, 8, 8, 11, and 21 years of age)	FMT	*Bacteroides, Barnesiella, Parabacteroides, Sutterella, Parasutterella, Clostridiales*, and *Erysipelotrichales* were most altered	Improved behavioral symptoms significantly of ASD children, with the exception of 21 years old subjects	–
Kang et al. (2017)	Cohort study of 18 ASD patients aged 7–16 years	MTT	Increased the diversity of bacteria in their gut, with the increased abundance of *Bifidobacterium*, *Prevotella*, and *Desulfovibrio*. And both of these changes persisted after treatment stopped (for 8 weeks)	Improved behavioral symptoms significantly of ASD patients (for 8 weeks)	GI symptoms improved (for 8 weeks)
Zhao et al. (2019)	Randomized, double-blind, controlled study of 48 ASD patients	FMT	Decreased the abundance of *Bacteroides fragilis*, and the gut microbiota of ASD patients gradually transferred to a healthy state. Changes of CARS were negatively correlated with *Coprococcus*	Decreased the CARS scores of the FMT group by a statistically significant 10.8% compared with a 0.8% decrease in the control group after the first FMT (F1), and still decreased slightly after the second FMT (F2)	Notable differences were also shown on GSI scores (*P* < 0.05) at F1 time point. 7 (29.2%) patients in FMT group reported adverse events such as fever, allergy and nausea, but all of them were mild, transient

*FMT, Fecal microbiota transplant; MTT, Microbiota Transfer Therapy; CARS, Childhood Autism Rating Scale; GSI, Gastrointestinal Severity Index; GI, gastrointestinal.*

In response to this, Kang et al. (2017) developed a modified FMT protocol (Microbiota Transfer Therapy, MTT) that consisted of 14 days of oral vancomycin treatment followed by 12–24 h of fasting bowel cleansing and then either oral or rectal administration of standardized human GM (SHGM) for 7–8 weeks. They found that 18 ASD children aged 7–16 years old not only improved their behavioral symptoms after treatment, but also increased the diversity of bacteria in their gut, with the increased abundance of *Bifidobacterium*, *Prevotella*, and *Desulfovibrio*. All of these changes persisted for at least 8 weeks after treatment ended. Furthermore, after 2 years of follow-up, the results showed that the ASD patients treated with MTT maintained a high diversity of gut bacteria and abundance of *Bifidobacterium* and *Prevotella*, and most of the improvement in gastrointestinal symptoms was also maintained. Importantly, behavioral symptoms were kept improved after 2 years of MTT treatment (Kang et al., 2019). The long-term effectiveness of this study shows that this treatment can maintain the remodeling of the gut of ASD patients, make their gut micro ecological system to achieve a healthy balanced state, and then improve the condition and the behavior level. The series of results confirmed an important role of overall gut microbes rebalancing during the process of intervention, which we believe to be an alternative and promising new approach for the treatment of GM dysbiosis in ASD.

## Conclusion

This review summarizes the therapeutic interventions for ASD based on gut microbiome, including dietary therapy, antibiotic therapy, probiotic and prebiotic intervention, and microbial transfer therapy. By evaluating the changes of microflora and disease characterization in the intervention process of these methods, we proposed that probiotics and prebiotics intervention methods have good efficacy and high safety. Furthermore, through our summary of probiotic intervention studies, we discovered that *Lactobacillus*, particularly *Lactobacillus plantarum*, may play important roles in improving anxiety and social behavior symptoms in ASD children. In the study of mice, researchers found that *L. reuteri* may be of great significance in improving the social behavior of ASD, while *Bacteroides fragilis* is of great significance in improving anxiety. Therefore, we believe that there may be some probiotics that can specifically improve the different behavioral symptoms of ASD. Future studies of single probiotic interventions should focus on the mechanisms with which the corresponding behavioral symptoms are influenced.

At present, there is a lot of evidence implying that the intestinal microbiota of autistic children is specific. However, due to the few studies based on GM, there are few subjects, large regional differences and inconsistent sequencing methods. It is difficult to propose a broad and effective ASD intervention method based on the modulation of GM. Moreover, although the effectiveness of mixed probiotic reagent, prebiotic reagent, mixed reagent of prebiotics and probiotics, and MTT emphasizes the importance of the overall balance of gut microbial system. The specific biological mechanism of these interventions is not clear, which is also a major problem in the development of corresponding interventions. Therefore, we believe that evaluating the internal biological mechanism between microbiota change and behavioral symptom improvement from the perspective of intervention may be the first concern of researchers.

Although many studies have discussed the characteristics of GM in ASD, there are few studies to supplement the corresponding probiotics for intervention according to the characteristics of GM in ASD patients. It is known that Delpro^®^, Del-Immune V^®^, VISBIOME, Vivomixx^®^, B-GOS, and some other broad probiotic supplements, are not work for every ASD child. However, as a special group, the GM of ASD patients is significantly different from that of healthy people or patients with other diseases. Therefore, subsequent intervention should develop specific probiotic and prebiotic reagents according to the characteristics of GM of ASD patients, and even develop corresponding personalized treatment schemes. Meanwhile, further research is still needed to prove the effectiveness and safety of probiotic and prebiotic therapy in the future.

Moreover, it is worth exploring that at present, researchers have different views on the association between GM and autism, including whether the microbiome differences found in the intestines of autistic children are due to their limited/specific dietary preferences related to the diagnostic characteristics of autism, or the reasons for their behavioral symptoms. The reason for this controversy may be that the internal mechanism of GM affecting the central nervous system is hard to measure and unclear. Therefore, we believe that while studying the specific biological mechanism of microbial-gut-brain axis, future research could focus on the changes of GM and behavioral symptoms of ASD patients during the intervention to help us have a deeper understanding of the relationship between microbiota and ASD.

## Author Contributions

CL, X-DJ, and JX conceived the project. JR, CF, and WW carried out the searches and synthesis. CL, JR, and JX interpreted the findings. CL and JR drafted the manuscript. CL and X-DJ approved the manuscript. All authors have read and approved the manuscript.

## Conflict of Interest

The authors declare that the research was conducted in the absence of any commercial or financial relationships that could be construed as a potential conflict of interest.

## Publisher’s Note

All claims expressed in this article are solely those of the authors and do not necessarily represent those of their affiliated organizations, or those of the publisher, the editors and the reviewers. Any product that may be evaluated in this article, or claim that may be made by its manufacturer, is not guaranteed or endorsed by the publisher.
